# Discovering Temporal Patterns in Longitudinal Nontargeted Metabolomics Data via Group and Nuclear Norm Regularized Multivariate Regression

**DOI:** 10.3390/metabo10010033

**Published:** 2020-01-13

**Authors:** Zhaozhou Lin, Qiao Zhang, Shengyun Dai, Xiaoyan Gao

**Affiliations:** 1 Beijing Institute of Chinese Materia Medica, Beijing 100035, China; 2 School of Chinese Materia Medica, Beijing University of Chinese Medicine, Beijing 10029, China; zhangqiao@bucm.edu.cn; 3 Division of Chinese Materia Medica, National Institutes for Food and Drug Control, China Food and Drug Administration, Beijing 100050, China; daisy@nifdc.org.cn

**Keywords:** nontargeted metabolomics, longitudinal study, antipyretic effects, multitask learning, structural regularization

## Abstract

Temporal associations in longitudinal nontargeted metabolomics data are generally ignored by common pattern recognition methods such as partial least squares discriminant analysis (PLS-DA) and orthogonal partial least squares discriminant analysis (OPLS-DA). To discover temporal patterns in longitudinal metabolomics, a multitask learning (MTL) method employing structural regularization was proposed. The group regularization term of the proposed MTL method enables the selection of a small number of tentative biomarkers while maintaining high prediction accuracy. Meanwhile, the nuclear norm imposed into the regression coefficient accounts for the interrelationship of the metabolomics data obtained on consecutive time points. The effectiveness of the proposed method was demonstrated by comparison study performed on a metabolomics dataset and a simulating dataset. The results showed that a compact set of tentative biomarkers charactering the whole antipyretic process of Qingkailing injection were selected with the proposed method. In addition, the nuclear norm introduced in the new method could help the group norm to improve the method’s recovery ability.

## 1. Introduction

With the advances of high throughput technologies, such as ^1^H nuclear magnetic resonance spectroscopy (^1^H-NMR) and mass spectrometry (MS), nontargeted metabolomics studies have been widely applied to elucidate biomarkers of diseases, to discover new therapeutic targets, to assign unknown gene function and to gain mechanistic insight into physiological processes in plants, yeast, bacteria, and mammals [1,2]. The high-dimensional nontargeted metabolomics data are typically analyzed with multivariate methods, including principal component analysis (PCA), partial least squares discriminant analysis (PLS-DA), and orthogonal partial least squares discriminant analysis (OPLS-DA) [3,4,5].

Some of the metabolomics studies belong to longitudinal study which is designed to compare changes over time under two or more experimental conditions. Thus, these metabolomics studies are helpful to elucidate the course of disease progression. Nonetheless, most of the nontargeted metabolomics data collected at a series of consecutive time points are separately analyzed at each time in a cross-sectional manner, i.e., one learns a model at every single time and then finds the union of all these points. However, this strategy splits the relevance across sequential times. Furthermore, the temporal nature of the data is not directly taken into account by common modeling methods. A direct consequence of ignoring the dynamic pattern is that temporal information is lost and the power to fully explore the time-related structure is damaged.

Even though the method adopted to analyze longitudinal nontargeted metabolomics data is very limited, a comprehensive review of potential methods appropriate for modeling dynamic patterns in time course metabolomics datasets has been provided by Smilde et al. [6]. Recently, approaches detecting temporal discriminatory metabolites have been proposed for extremely short time series cases. Maurice Berk et al. [7] proposed a statistical framework consisting of a smoothing spline mixed effect (SME) model and an associated functional test statistic for biomarker discovery in longitudinal metabolomics data. Sonja Peters et al. [8] used autocorrelation and goodness-of-fit of exponential curve-fitting to distinguish metabolites showing meaningful trends from those showing only random variations. However, these univariates coupled with unsupervised strategies lack the efficiency to explore the relationship among variables.

In addition to the methods directly using the dynamic character of each metabolite, multitask learning (MTL) methods can also be used to discover metabolites whose concentration variations are associated with metabolic status (or conditions). Zhang D et al. [9] developed a longitudinal variable selection (LFS) approach to jointly select features across multiple time points. For each time point, a sparse linear multivariate linear regression model was built. Coupled with an extra ‘group regularization’, the weights corresponding to the same feature across multiple time points were grouped together. The group regularization term allows the variables to be selected based on the strength of multiple time points jointly rather than only based on the contribution of variables in a single linear model. Nevertheless, the ‘group regularization’ may not thoroughly address the correlations at different time points. Jia-yu Zhou et al. [10] formulated the progression of clinical scores as a multitask regression problem by considering the prediction of cognitive scores at a single time point as a regression task. However, the plain temporal group Lasso regularization algorithm is not applicable for metabolomics data measured at sequential time points. Since it was developed to predict the cognitive score of selected patients at multiple time points using the data at baseline rather than classify the samples using data measured at multiple time points.

Generally, biomarkers characterizing specific physiological process should be detected over a long period. In this study, a novel structural regularized multivariate regression method was developed based on this assumption to efficiently discover biomarkers whose longitudinal profile showed significant differences between the intervention and control group. The regularization terms employed in the proposed approach make full use of the dynamic character of metabolite trajectories. Specifically, the group (*l*_2,1_ norm) regularization term ensures that all regression models at different time points share a common set of features. Meanwhile, the trace norm reserves the association patterns at consecutive time points. To demonstrate the efficiency of the proposed method, a comparison study between the proposed approach and the cross-sectional analysis strategy together with the LFS was performed on the yeast induced pyretic metabolomics data and a simulating data.

## 2. Results

The metabolomics dataset was used to demonstrate the powerless of cross-sectional method to deal with longitudinal data and test the performance of the proposed method. Sixteen infected rats were divided into two groups. Urine of each rat was collected at designed time points and measured by LC-MS. Finally, 1276 peaks were extracted for further analysis. The simulating data was generated to evaluate the variable recall ability of the two longitudinal methods. At each time point, 50 simulating samples were generated for both positive and negative class. Each sample had 1500 variables, 1300 of which are uninformative variables.

### 2.1. Cross-Sectional Analysis with PLS-DA

Since PLS-DA is commonly used in a nontargeted metabolomics study to identify tentative biomarkers, it is natural to use it directly in time course metabolomics studies. At each time point, the variables whose VIP score larger than 2 were retained, afterwards, the PLS-DA model was retrained on the retained variables. Table 1 shows the performance of these PLS-DA models. With the discriminative variables retained, an obvious improvement on the prediction performance of the PLS-DA model was achieved in terms of the ACC (accuracy), CE_AUC (conditional expected AUC) and DQ^2^ (discriminant Q^2^). From the DQ^2^, it can also be inferred that the samples that are located near the decision bound were reduced. In addition, CE_AUC performed similar to ACC, and both methods could not further discriminate the classification models after they reach one. Thus, only CE_AUC was used in the following analysis.

These results demonstrated experimentally the power of VIP-based variable selection method to discover discriminative metabolites. However, the biomarkers characterizing specific physiological process should be detected over a long period. More specifically, the tentative biomarkers selected at a certain time point should work well not only at the modeling time point, but the adjacent time points. Thus, the efficiency of the variables selected at each time point by the VIP based method were also tested on the other time points.

As is shown in Table 2, with the variables selected at the fourth hour, three new PLS-DA models were retrained on the other time points. However, all of the three new PLS-DA models performed worse than the models constructed with variables selected on their own time points. Additionally, two of the three PLS-DA models performed even worse than their corresponding full PLS-DA model. This result indicated that the variables selected at the fourth hour failed to characterize the antipyretic effect of QKL injection. In addition, neither the variables selected at 12 h nor the variables selected at 24 h could characterize the antipyretic effect of QKL injection. Nevertheless, the variables selected at the eighth hour presented acceptable results on all the four time points, except the PLS-DA model generated at 24 h with variables selected at 8 h. Above all, the variables selected on one time point were not guaranteed to work well on the adjacent time points, let alone a whole physiological process.

**Figure 1:**
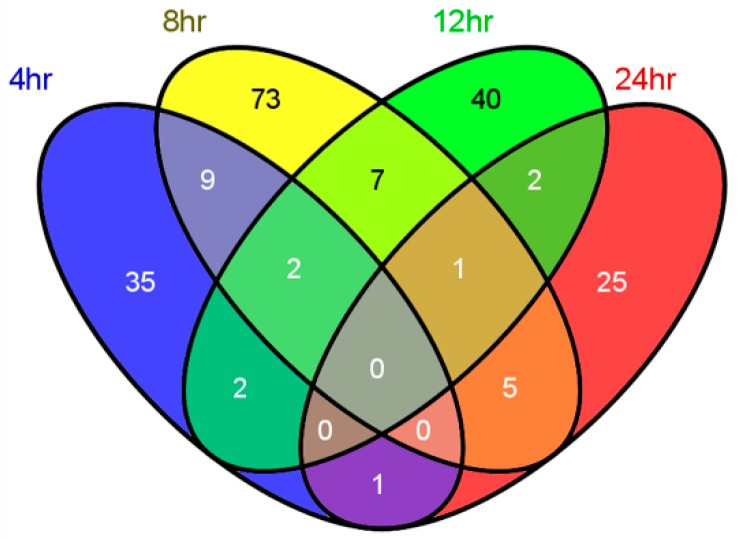
Venn diagram showing the relations between the features reported for each time point.

A straightforward strategy to select metabolites whose contribution are equally important to all the measurement of a specific physiological process is to use the metabolites adjacently selected at all of the four time points. However, as observed in Figure 1, there were no variables selected commonly for all the four time points. Among each variable set, most variables were specific to their modeling time points.

These results clearly illustrate the powerlessness of the cross-sectional analysis strategy. However, this does not mean that the antipyretic process can be separated into several individual stages, each with their own character.

### 2.2. Discovering Longitudinal Metabolomics Markers

To identify the metabolites’ work for the whole antipyretic process, LFS whose efficiency in finding temporal biomarkers had been experimentally demonstrated was used. The regularization parameter of LFS was optimized under the guidance of LPOCV error. The results of applying LFS with the optimal parameter γ = 0.1 on the antipyretic data were listed in Table 3. Compared with the full PLS-DA model, the prediction performance of LFS at all of the four time points estimated by LPOCV procedure were improved in terms of conditional expected AUC. Moreover, a considerably larger improvement on the prediction performance at all the sampling points were obtained in terms of DQ^2^ except for the 24 h.

**Figure 2:**
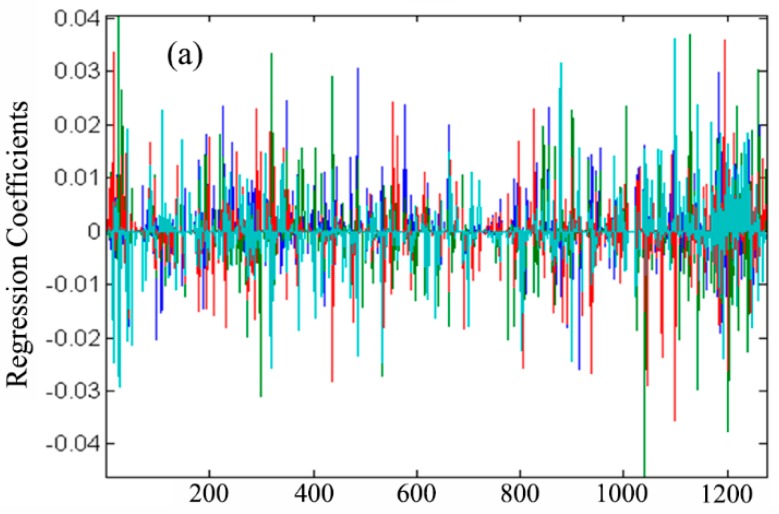

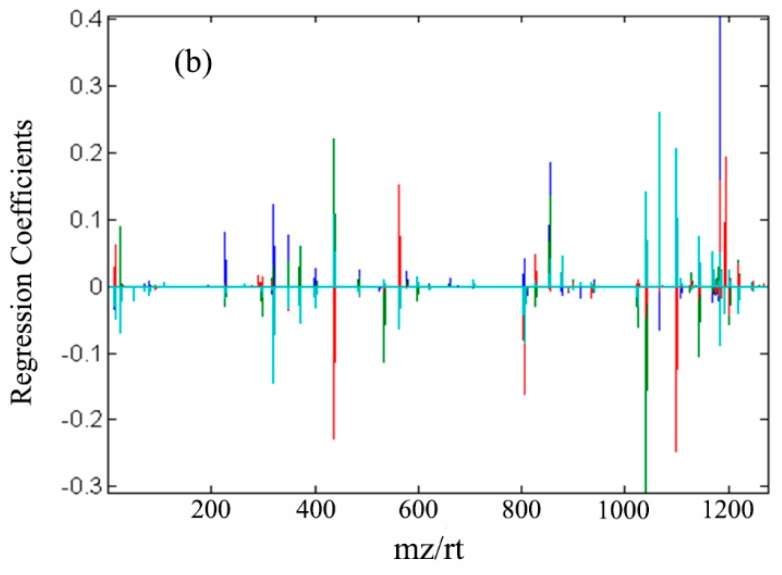
Overlay plot of regression coefficients. (**a**) The coefficients of LFS; (**b**) the coefficients of GNNR. Different colors correspond to different time points.

Figure 2a shows the regression coefficients of LFS. As expected, some of the regression coefficients at all of the four time points were exactly zero. Therefore, the LFS algorithm can select tentative biomarkers automatically. However, the number of variables whose regression coefficient deviate from the zero line is much more than the following biological interpretation and experimental validation procedures can afford. Thus, the variables whose median absolute value of regression coefficients is larger than 10^−2^ were marked as a discriminative variable.

To obtain a sparse tentative biomarker set, a new structural regularization method was proposed. It inherited all the sound priorities of LFS and took the similarity of the models between adjacent time points into account by using the nuclear norm regularization.

Each regularization parameter pair (γ_1_, γ_2_) in the cross-product of γ_1_ $\in \left\{10^{-6},\ldots ,\;1,\ldots ,\;10^{3}\right\}$ and γ_2_ $\in \left\{10^{-6},\ldots ,\;1,\ldots ,\;10^{3}\right\}$ was used to train a multivariate regression model. For each model, the statistics were estimated by the LPOCV procedure. Actually, the parameter tuning process falls into the domain of multiple-criteria decision analysis. Therefore, the minimax regret criterion was employed to determine the optimal regularization parameter pair. Finally, the regularization parameters (γ_1_, γ_2_) were set to be (10^−3^, 10^−6^).

As shown in Table 3, the results of applying GNNR on the metabolomics data were improved considerably in comparison with the full PLS-DA model in terms of DQ^2^. However, not all the results of GNNR at all four different time points were better than that of LFS. Specifically, the prediction results characterized by the condition of expected AUC indicated that there was no improvement on the model built by GNNR, since it performed slightly worse than LFS on the data obtained at the eighth and twelfth hour. Nevertheless, these results demonstrated that the extra regularization imposed on the regression coefficient matrix to account for the similarity between adjacent models did not damage the prediction ability of GNNR.

An overlay plot of the regression coefficients of GNNR on four time points is shown in Figure 2b. It can be observed that there is no difference among the variables selected at each time point. This result demonstrated that the antipyretic effect of QKL injection could be characterized by a common and compact variable set. The variation of the regression coefficient contributed significantly to the changes in physiological status. From the objective function of GNNR and LFS, it can be concluded that the association patterns at consecutive time points are critical to finding metabolites charactering the whole antipyretic process.

**Figure 3:**
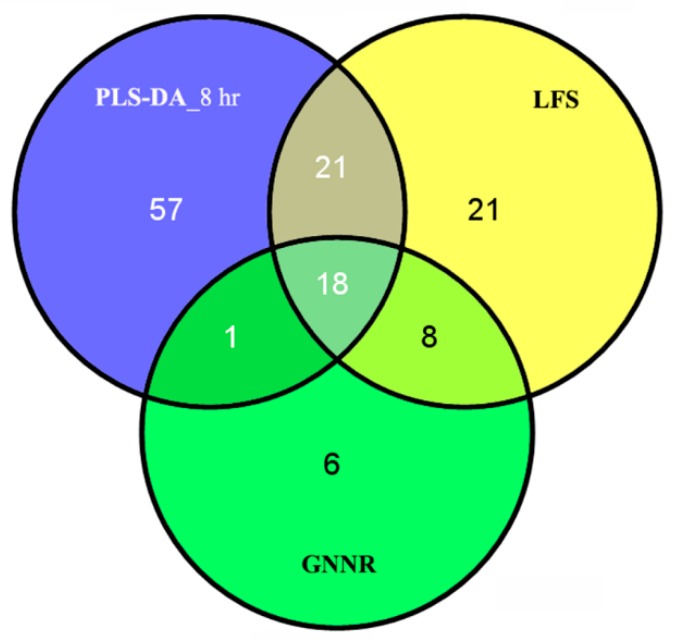
Venn diagram comparing the overlap between the features reported by each method.

To further explore the difference among the variables selected by the above three methods, a Venn plot showing their relationship was given in Figure 3. Most of the variables selected by GNNR could be selected by the other two methods, but not vice versa. In addition, although each learning method had their own character, eighteen variables were selected by all the three methods. Since it was believed that a common but unknown subset of variables is critical to the antipyretic process. In conclusion, all the results obtained demonstrated that by adding nuclear norm into the objective function, the predictive metabolomics markers characterizing the antipyretic process of QKL injection could be selected by the proposed GNNR algorithm.

### 2.3. The Recovery Ability of the Two Longitudinal Methods on Simulating Data

The simulating data is a cube of size 100 × 1500 × 4. Namely, on each of the four time points, a data matrix was generated. Each data matrix contains 100 samples, and each sample has 1500 variables. If any of the 1300 uninformative variables were selected from the entire variable set, it would be considered as false recovery. Actually, it is hard to avoid the false recovery considering the large noise background.

To select the optimal parameter or parameter combination, the statistics including DQ^2^, CE_AUC, and the number of misclassification samples (NMC) were estimated by the stratified 10-fold cross validation, which is a generalization of LPOCV. It first divides the observations into *k* disjoint fold in each class (group), one fold from each class were randomly left as test samples. Thus, each inner test set has roughly equal size and roughly the same class proportions as in the full sample set.

For LFS, the multivariate regression model with γ equal to 1 presented the highest DQ^2^ on all the four time points. While, from the results of NMC and CE_AUC, it was found that the prediction performance of the regularized model remained unchanged until the value of γ reach 10^3^. Since their powerless to guide model selection, NMC and CE_AUC were not used in the following analysis.

**Figure 4:**
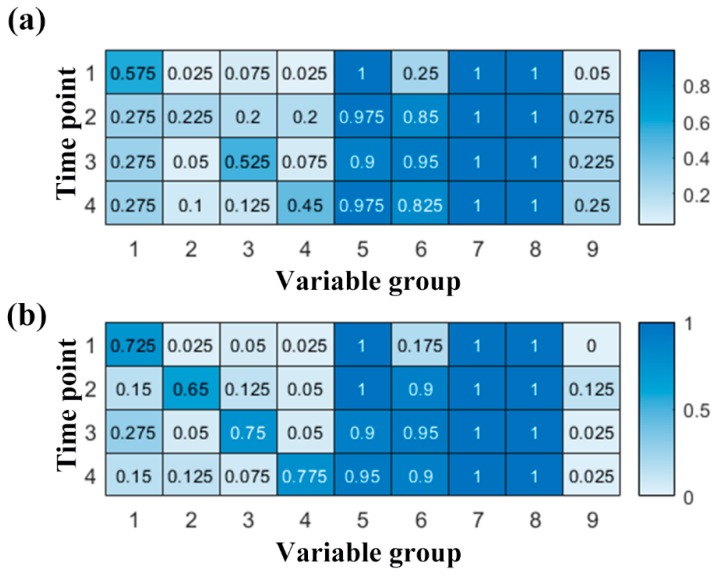
Heat map of the recovery ratio for LFS (**a**) and GNNR (**b**). The variable group 9 denotes the false recovered uninformative variables; the variable group 1~4 corresponds to type A’ to D’ of Figure 5b and the variable group 5~8 corresponds to type A to D of Figure 5a, respectively. The light blue cells represent the false discovery ratio except for the second group on time point two of the subplot (**a**). While the dark blue table cells show the true discovery ratio.

**Figure 5:**
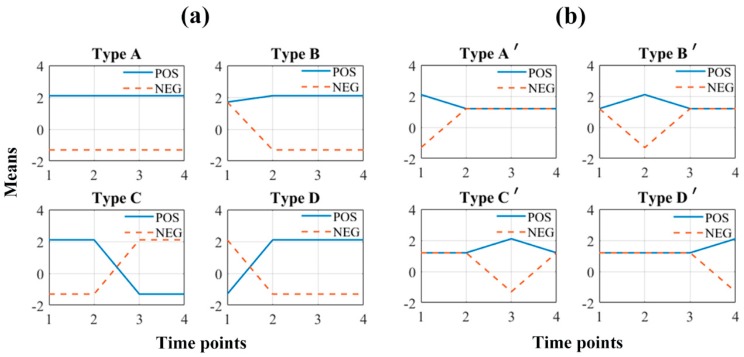
Two groups of profiles formed by linking the mean values on each time point. (**a**) The variables detected on a long period or more than one time point; (**b**) the variables detected on one single time point. POS in the legend means the variables were generated for the positive class, and NEG stands for the negative class.

Ideally, the redundant but uncorrelated discriminative variables should all be recovered from the noisy simulating data. The recovery ratio, i.e., the ratio between the number of variables selected by the variable selection method and the number of the entire variable set, was calculated. The group norm of LFS could select variables based on the strength of multiple time points jointly, but this did not mean the contribution of one specific variable for the model of different time points should be equal. Thus, the variables were sorted according to the amplitude of regression coefficient on each time point, separately. Then, the first *n* variables were selected. For the coefficient of the first time point, *n* was set to 160, and for the other time points *n* was set to 200. Figure 4 showed the ratio of variables belonging to the category designed in Section 4.3. Most of the variables corresponding to the profiles of Figure 5a were recovered, but the false recovery ratio of the variables specific to each time point were about 50%. Some variables even belonged to the uninformative background (Variable group 9).

The optimal regularization parameters of GNNR were determined by DQ^2^. In the optimal model, the regularization parameter for the group norm was 0.1, the weight parameter for the trace norm was 10^−5^. To further illustrate the difference between the two longitudinal methods, the coefficient matrix of GNNR were also analyzed such as LFS. As shown in Figure 4b, although GNNR could not recover the entire discriminative variables specific to each time point, the recovery ratio was improved when compared with the LFS. In addition, it was observed that the false recovery ratio was reduced obviously. Moreover, the number of uninformative variables were also suppressed by GNNR.

## 3. Discussion

Latent variable models, e.g., PLS-DA and OPLS-DA, have been widely used in nontargeted metabolomics studies to select biomarkers characterizing the affected metabolic process. One of the inherent advantages of these methods is that the absolute value of regression coefficient is positive relative to the variable’s contribution in the model. This character facilitates the interpretation and understanding of the resulted model. Several methods have been developed to rank metabolites based on the coefficients of model.

However, every coin has two sides. The reasonability of using these methods in some cases is suspectable. One of such cases is to use the latent variable method on data measured at consecutive time points. To illustrate experimentally the negative effect of information loss in cross-sectional analysis, variables selected on one time point were used to retrain the model on the other three time points. The generalized error of all the three new models were estimated separately by LPOCV. The results showed that the new models performed worse than the models constructed with variables selected on their own time points. On the contrary, the variables selected by LFS and GNNR could produce a model working for all time points. This result demonstrated the necessity of analyzing longitudinal data together to find biomarkers characterizing the physiological process. The difference between the sparseness of the final variable set of GNNR and LFS could be attributed to the nuclear norm. Although nuclear norm introduced in the GNNR could not directly influence the number of nonzero entries in a vector, minimizing the rank of the regression matrix might force certain variables to be shrunk towards zero, and assist the *l*_1_-norm to limit the number of variables having nonzero value.

The relationship among real data can only be perceived but cannot be precisely described. Thus, the simulating data including variables showing specific trajectory were generated to investigate the recovery ability of the two longitudinal methods in this study. Results showed that most of the variables showing certain trajectories could be selected by the two longitudinal methods, while the nuclear norm introduced in GNNR could help the group norm to recall more variables working for only one time point. Although increasing the regularization parameter of group norm for both methods could produce a more compact variable set, and the number of misclassified objects would remain undamaged, the DQ^2^ would decrease seriously. In addition, the results also demonstrated that the commonly used accuracy and NMC could not effectively discriminate the model in some cases. Therefore, the sparseness of the regularization method should be pursued under the guidance of effective and discriminative statistics.

The models trained using LFS or GNNR on MS data were worse than the PLS-DA models trained individually on each time point. There are two factors that might interpret these phenomena. The first one is that the regularized multivariate regression generally work worse than the PLS on data whose number of variables is far larger than the number of samples. The other one is that the trace norm may be too rigorous for the pyretic process. Applying the norm regularization on two consecutive time points might alleviate the problem. Thus, future work will focus on these two directions.

## 4. Materials and Methods

### 4.1. Methods

Let $\chi =\{X_{1},X_{2},\ldots ,X_{T}\}\in {\mathbb{R}}^{d\times n\times T}$ corresponds to the measurements taken at *T* consecutive time points, where ***X****t* represents the metabolomics data collected at the *t-*th (1 ≤ *t* ≤ *T*) time point with *d* features and *n* samples. Denote $y_{t}\in {\mathbb{R}}^{n}$ as the corresponding target outputs at the time point *t*. Consider that the problem of identifying discriminatory metabolites characterizing each condition can be formalized as a classification one. Therefore, $y_{t}\in {\left\{-1,1\right\}}^{n}$.

#### 4.1.1. Longitudinal Feature Selection

The longitudinal feature selection (LFS) algorithm was proposed by Zhang D. et al. [9] to jointly select features across multiple time points. At each time point, a sparse linear regression model relating the metabolomics data to the categorical responses is trained. The group regularization imposed on the regression coefficient ***β***s across *T* time points forces the weightings of the same feature across multiple time points grouped together and allows for selection of features based on the strength of multiple time points jointly. The group norm regularized least squares function is formulated as:

(1) $$\underset{B}{\min}\frac{1}{2}\sum_{t=1}^{T}{\left\|y_{t}-X_{t}\beta_{t}\right\|}_{2}^{2}+\lambda \sum_{j=1}^{d}{\left\|\beta^{d}\right\|}_{2}$$

where, $B=[\beta_{1},\beta_{2},\ldots ,\beta_{T}]\in {\mathbb{R}}^{d\times T}$, and $\beta^{d}$ is its *d*-th row vector. The regularization parameter λ balances the relative contribution of the prediction error and the sparsity of the linear models. The parameter λ can be optimized by leave-one-pair-out cross validation (LPOCV) [11]. Analogously to leave one out cross validation, each possible positive–negative pair of samples is left out of at a time from the training set. In pooling, the predictions made in each cross-validation are pooled into one set and the statistics are calculated from it.

Due to the parsimony property of *l*_2,1_-norm in the above objective function, it produces a regression coefficient matrix **B** with elements in some rows being exactly all zeros. Therefore, only those metabolites whose regression coefficient is greater than zero are selected for further identification.

#### 4.1.2. Group and Nuclear Norm Regularization

The key of finding temporal patterns in multitask learning is to exploit the intrinsic relatedness among tasks. However, although the group sparse regularization forces the metabolites with common influence across all the time points to be retained in the final model, the association of consecutive time points are ignored. Furthermore, the association patterns (models) at two consecutive time points tend to be similar [10,12], and the coefficient matrix should be of low rank. Thus, the nuclear norm is added in the objective function to approximate the rank function. Finally, the structured sparsity-inducing norms, including *l*_2,1_-norm and nuclear norm, are introduced into the objective function of multivariate regression. The resulting model is expected to reveal the temporal change of metabolites. The group and nuclear norm regularization (GNNR) model is constructed by solving the following optimization problem:

(2) $$\underset{B}{\min}\ell (B)+\gamma_{1}\sum_{j=1}^{d}{\left\|\beta^{d}\right\|}_{2}+\gamma_{2}{\left\|B\right\|}_{*}$$

with,

$$\ell (B)={\left\|B{⊗}_{1}\chi^{T}-Y\right\|}_{F}^{2}$$

$${\left\|B\right\|}_{*}=\sum_{i=1}^{\min\{m,n\}}\sigma_{i}=trace(\sqrt{B^{T}B})$$

where, ℓ(B) is the loss function, ${\left\|\cdot \right\|}_{*}$ denotes the trace (or nuclear) norm of a matrix.

Despite its sound properties, the objective function in Equation (2) is a convex but nonsmooth problem. An iteratively reweighted method [13] is employed to solve the nonsmooth regularization problem. The derivative of Equation (2) is taken with respect to $\beta_{t}$ and set to 0:

(3) $$2X_{t}X_{t}^{T}\beta_{t}-2X_{t}y_{t}+2\gamma_{1}D\beta_{t}+2\gamma_{2}\tilde{D}\beta_{t}=0$$

where $D(j,j)=\frac{1}{2}{\left(\sum_{t=1}^{T}b_{t}^{j}\right)}^{-1/2}$ and $\tilde{D}=\frac{1}{2}{\left({\mathrm{BB}}^{T}\right)}^{-1/2}$.

Rewriting Equation (3):

(4) $$(X_{t}X_{t}^{T}+\gamma_{1}D+\gamma_{2}\tilde{D})\beta_{t}=X_{t}y_{t}$$

Then, the equation below is derived.

(5) $$\beta_{t}={\left(X_{t}X_{t}^{T}+\gamma_{1}D+\gamma_{2}\tilde{D}\right)}^{-1}X_{t}y_{t}$$

For each t∈[1,T], *β_t_* can be calculated by Equation (5). Meanwhile, both *D* and $\tilde{D}$ are dependent on **β**, thus the regression coefficient can be estimated by an iterative algorithm, which is summarized as follows:

1. For data: $\chi \in {\mathbb{R}}^{d\times n\times T}$, $Y\in {\left\{-1,1\right\}}^{n\times T}$

Initialize $B^{\left(0\right)}\in {\mathbb{R}}^{d\times T}$ using the regression results at each individual time point.

2. Calculate the diagonal matrix D, where the *k*-th diagonal element is computed as $D(j,j)=\frac{1}{2}{\left(\sum_{t=1}^{T}b_{t}^{j}\right)}^{-1/2}$.

3. Calculate $\tilde{D}=\frac{1}{2}{\left(BB^{T}\right)}^{-1/2}$.

4. Update $\beta_{t}$ by $\beta_{t}={\left(X_{t}X_{t}^{T}+\gamma_{1}D+\gamma_{2}\tilde{D}\right)}^{-1}X_{t}y_{t}$.

Repeat 2~4 until converges.

Finally, $B=[\beta_{1},\beta_{2},\ldots ,\beta_{T}]\in {\mathbb{R}}^{d\times T}$.

The structural regularization terms imposed on the multitime point regression model endow GNNR with the property of automatic variable selection. Thus, the metabolites with regression coefficient larger than 0 were marked as tentative biomarkers. The regularization parameters, i.e., γ of LFS and γ_1_ and γ_2_ of GNNR, were tuned in the range of $\left\{10^{-6},\ldots ,\;1,\ldots ,\;10^{3}\right\}$.

#### 4.1.3. PLS-DA

The commonly used partial least squares discriminant analysis (PLS-DA) is a variant of the classical partial least squares regression technique [14], which relates the metabolic data (e.g., **X**_t_) to an integer $y_{t}\in {\left\{-1,1\right\}}^{n}$ designating the class of the sample. The discriminatory metabolites are selected on the basis of VIP scores, since the regression coefficients of the PLS-DA method lack the scarcity property. The VIP score of each variable is calculated as [15]:

(6) $${\mathrm{VIP}}_{j}=\sqrt{p\overset{h}{\underset{k=1}{\sum }}(SS(b_{k}t_{k}){\left(\frac{w_{jk}}{\left\|w_{k}\right\|}\right)}^{2})/\overset{h}{\underset{k=1}{\sum }}SS(b_{k}t_{k})}$$

where, $SS(b_{k}t_{k})=b_{k}^{2}t_{k}^{t}t_{k}$, *k* = 1, 2… h, *p* is the number of columns of **X***t*; *w_jk_* is the loading weight of the *j-th* variable in the *kth* component; *b_k_*, *t_k_*, and *w_k_* are the *k-th* elements or vectors of ***b***, **T**, and **W** respectively. The *VIP* score quantifies the influence of each variable over all *h* latent variables and categorical ***y***_t_.

#### 4.1.4. Performance Estimation

The conditional expected area under the ROC curve (CE_AUC) [11] measures how well a prediction function learned from a certain dataset will do on future samples. However, the probability distribution used to calculate the AUC metrics can never be directly accessed, some estimation method can be used instead, such as the one obtained from cross-validation. Therefore, LPOCV is used for the AUC calculation, since it avoids many of the pitfalls associated with the pooling or averaging techniques. Each possible positive–negative pair of samples is left out individually from the training set. The AUC performance can be calculated using the following formula:

(7) $$\hat{A}(X,fz)=\frac{1}{\left|X_{+}\right|\left|X_{-}\right|}\sum_{x_{i}\in X_{+}}\sum_{x_{j}\in X_{+}}H(f_{-\{i,j\}}(x_{i})-f_{-\{i,j\}}(x_{j}))$$

where *X* is a sequence of examples,$f_{-\{i,j\}}$ denotes a classifier trained without the *i*-th and the *j*-th samples, $X_{+}\subset X$ and $X_{-}\subset X$ denote the positive and negative examples in *X*, respectively.

The discriminant Q^2^ (DQ^2^) is first introduced by Johan A. et al. [16] as an improvement for the Q^2^ which is commonly used in the validation of classification models. It does not penalize class predictions beyond the class label value. DQ^2^ is defined as:

(8) $$DQ^{2}=1-\frac{PRESSD}{TSS}$$

where, $\underset{Class1}{PRESSD}={\sum_{{\hat{y}}_{i}<1}\left(y_{i}-{\hat{y}}_{i}\right)}^{2}$, and $\underset{Class-1}{PRESSD}={\sum_{{\hat{y}}_{i}>-1}\left(y_{i}-{\hat{y}}_{i}\right)}^{2}$.

*TSS* denotes total sum of squares of the response vector **y**.

The prediction error is disregarded when the class prediction is beyond the class label. Specifically, when the prediction is above 1 for class 1 samples or when the prediction is below –1 for class –1 samples, the prediction error for that sample is ignored.

Although the models of different time points were trained simultaneously using the longitudinal methods, the prediction error did not vary synchronously along regularization parameters. Therefore, the minimax regret criterion was used to determine the optimal regularization parameters. It first created the regret matrix by calculating the distance from optimum for each time point and then found the parameters with the minimum maximum regrets.

### 4.2. Sample Background and Preparation

The proposed algorithm was evaluated using the metabolomics data reported in [17]. To reveal the antipyretic mechanism of Qingkailing (QKL) injection in yeast-induced pyrexia rats, two groups of yeast-induced pyrexia rats were used. Each group has eight samples. One group of model rats was treated by QKL injection, while the other group was subcutaneously injected with an equal volume of 0.9% saline. Urine samples from the pyrexia model group (PG) and the group treated by QKL injection (TG) were collected before yeast was injected and at a series of time points after QKL injection, including 4, 8, 12, 24, 36, 48, and 72 h. Urine samples collected before being injected with yeast were used as a baseline. The urine samples were centrifuged at 14,000 rpm for 10 min at 4 °C. The supernatants were collected and stored at −20 °C. After being thawed at room temperature, the supernatant was diluted. A 2 µL sample was injected into a UPLC Q-TOF/MS system for analysis.

The UPLC-MS chromatograms of all samples were converted to .cdf format. The R package XCMS was employed for peak discrimination, filtering, and alignment [18]. XCMS parameters were set as default except for the following: Filtration and peak identification: centWave method, ppm = 15, peak width = c (5, 20) s, snthresh = 6, prefilter peaks = 3, prefilter intensity = 1000; retention time correction: Obiwarp method; alignment: Mzwid = 0.015, bw = 2. The parameter “prefilter intensity” was set to 1000 to facilitate the following structure identification. Moreover, the redundant variables originating from the same metabolite, such as the fragments, isotope compositions, and adduct ions, were removed using the R-package CAMERA [19,20]. Finally, 1276 obtained peak variables were imported into MATLAB.

Since the rats were randomly divided into PG and TG groups and underwent the same treatment at baseline, the UPLC-MS data collected at baseline were nondiscriminable and were deleted. Moreover, the rectal temperature of rats from the PG and TG groups showed distinct differences before 36 h only (Figure 1 of [17]). Therefore, only the data collected at the time points 4, 8, 12, and 24 h were used.

### 4.3. Simulating Data

The simulating data contains 100 objects, 50 for each class. Each object was assumed to be measured at four time points. At each time point, 40 variables were randomly generated from the normal distribution with mean defined at the corresponding point of each profile in Figure 5. An object was labeled positive if it was generated from the means defined for the positive class. The normal distributions used to generate the simulating variables were *N* (2.1, 1.5^2^), *N* (1.7, 1.5^2^), *N* (1.2, 1.5^2^), and *N* (−1.3, 1.5^2^). The value 2.1 and −1.3 were chosen according to the range of metabolomics data preprocessed in the above section. The value 1.7 was applied to represent undistinguishable variables, and the value 1.2 was set to mimic uninformative variables. The uninformative variables referred to the fragments responding rarely to the stimulation in metabolomics experiments. These types of variables are common in practical data. Thus, 1180 variables were randomly generated for each object. While, the undistinguishable variables indicated the fragments changing in the same direction, but based on them the positive and negative samples cannot be distinguished. Finally, the simulating data was arranged to a cube of size 100 × 1500 × 4.

### 4.4. Software

Raw LC-MS data files were directly processed by the open source XCMS package under R statistical software (version 3.1.0) to carry out peak discrimination, filtering, and alignment. The PLS-DA model and the proposed GNNR learning model were constructed using the in-house scripts in MATLAB 7.8 (Math Works, Natick, MA, USA). The LFS method was implemented based on the algorithms in SELP toolbox [21].

## 5. Conclusions

Longitudinal nontargeted metabolomics studies are helpful to elucidate the course of disease progression, which is of great value for both scientific research and clinical practice. In this article, a novel structural regularization method was developed to select metabolites whose longitudinal profile show significant differences between the intervention and control group. The metabolomics data investigating the antipyretic effects of Qingkailing injection in rats was used to evaluate the effectiveness of the proposed method. The results demonstrated that the prediction performance of LFS was improved in comparison with the full PLS-DA model, but the sparsity of LFS was limited. Unfortunately, the cross-sectional analysis strategy did not work on this particular data. Nonetheless, the nuclear norm introduced into the objective function of GNNR did not damage its prediction performance, but produced a compact set of potential biomarkers, which characterizes the entire antipyretic process of Qingkailing injection. A simulating data was further used to investigate the recall ability of the two regularization methods. Results demonstrated that the nuclear norm introduced in the GNNR is helpful to recall variables working for only one time point. While being potentially promising, the GNNR method needs to be further validated with other longitudinal metabolomics studies. This is currently being pursued.

## Figures and Tables

**Table 1:** Figures of merit of PLS-DA (Partial Least Squares Discriminant Analysis) models established by LPOCV (leave-one-pair-out cross validation) before and after variable selection.

	Full PLS-DA			N of Vars.	Reduced PLS		
Time Points	CE_AUC	ACC	DQ^2^	-	CE_AUC	ACC	DQ^2^
4 h	0.7814	0.70	0.3769	49	1	1	0.9857
8 h	0.7501	0.72	0.1990	97	1	1	0.9150
12 h	0.7968	0.87	0.0553	54	1	1	0.7981
24 h	0.8749	0.88	0.6542	34	1	1	0.9876

**Table 2:** A summary of the prediction performance of PLS-DA (Partial Least Squares Discriminant Analysis) models retained on adjacent time points after variable selection.

	CE_AUC				DQ^2^			
Time Points	4 h	8 h	12 h	24 h	4 h	8 h	12 h	24 h
4 h *	1	0.9344	0.7808	0.8576	0.9857	0.6122	−0.3148	0.4112
8 h *	0.9344	1	0.9088	0.8768	0.6969	0.9150	0.3671	0.3085
12 h *	0.7360	0.8	1	0.9536	−0.1339	−0.3079	0.7981	0.7672
24 h *	0.6720	0.7967	0.9216	1	−1.0532	0.1240	0.5987	0.9876

The * signifies that the variables were selected based on the model trained on data of these time points.

**Table 3:** Comparisons between the proposed GNNR (Group and Nuclear Norm Regularization) and LFS (Longitudinal Feature Selection) in terms of condition expected AUC (Area Under Curve) and discriminant Q^2^.

Methods	Metrics	4 h	8 h	12 h	24 h
LFS	CE_AUC	0.8	0.7808	0.8448	0.9088
	DQ^2^	0.4074	0.3658	0.5024	0.6301
GNNR	CE_AUC	0.8768	0.7168	0.8256	0.9216
	DQ^2^	0.4614	0.2843	0.5112	0.6740
